# Cardiovascular risk stratification in inflammatory-driven conditions: how far have we come?

**DOI:** 10.3389/fcvm.2026.1601800

**Published:** 2026-01-27

**Authors:** Gustavo L. R. Silva, Andrei C. Sposito, Otavio Coelho-Filho, Thiago Quinaglia

**Affiliations:** Discipline of Cardiology, Department of Medicine, Faculty of Medical Science, State University of Campinas, Campinas, Brazil

**Keywords:** cancer, cardiovascular risk, HIV, inflammation, rheumatic and autoimmune disease

## Abstract

Atherosclerotic cardiovascular disease (ASCVD) risk stratification has predominantly relied on conventional risk factors such as hypertension, hyperlipidemia, diabetes mellitus, smoking, and family history of premature ASCVD. These features, while important, often fail to capture the complex interplay of contributors to ASCVD. Inflammation is involved in the initiation, progression, and destabilization of atherosclerotic plaques. However, it has not been consistently included in cardiovascular risk stratification tools which are crucial to trigger interventions and prevent events. Thus, there has been a growing interest in incorporating inflammatory biomarkers into ASCVD risk assessment. Two researchers entered the search terms: “cardiovascular” AND “risk stratification” AND “inflammation” AND alternately the following aditional terms: “primary prevention” OR “secondary prevention” OR “residual risk” OR “inflammatory disease” in the PubMed and Open Evidence platforms. In total, 25 studies were analysed and discussed: 1 meta-analysis, 15 cohort studies, 9 cross-sectional studies. Other studies were included to support the evidence and provide context. Inflammatory-driven conditions can lead to a 24% to almost three-fold increase in cardiovascular mortality risk compared with the general population even in the absence of traditional risk factors. Furthermore, stratification of inflammatory-driven risk is largely unexplored in primary and secondary (residual risk) prevention. Traditional scores are least effective in the intermediate-risk group where a proportion of over 50% of individuals are misclassified. In secondary prevention, estimates show that 38% of individuals persist with elevated high-sensitivity C-reactive protein and these patients exhibit higher all-cause mortality, myocardial infarction and stroke rates at one year. In this review we aim to gather and appraise studies presenting alternatives to traditional risk assessment that consider the inflammatory contribution to cardiovascular events in primary and secondary prevention levels, as well as, in populations living with inflammatory-driven diseases.

## Introduction

Atherosclerotic cardiovascular disease (ASCVD) remains a leading cause of morbidity and mortality worldwide, posing a significant burden on healthcare systems and individual well-being (1, 2). Traditionally, ASCVD risk stratification has predominantly relied on conventional risk factors such as hypertension, hyperlipidemia, diabetes mellitus, smoking, and family history of premature ASCVD. These factors, while important, often fail to capture the complex interplay of factors contributing to ASCVD (3, 4). The addition of novel markers to these tools has arguably become unavoidable to improve their calibration and discrimination. Systemic inflammation plays a pivotal role in the initiation, progression, and destabilization of atherosclerotic plaques, ultimately leading to cardiovascular events. Inflammation promotes endothelial dysfunction, lipid accumulation within the arterial wall, and plaque instability (5). Conversely, oxidized LDL cholesterol within the arterial wall and advanced glycation end-products (AGEs), for instance, can trigger and amplify inflammatory responses, creating a self-perpetuating cycle that accelerates disease progression (6, 7). Moreover, risk reductions observed in trials of IL-1β inhibition and of colchicine are at least as large in magnitude as those seen associated with adjunctive lipid-lowering agents among patients already taking a statin (8).

This understanding has spurred a growing interest in incorporating inflammatory biomarkers into ASCVD risk assessment, especially within the context of inflammatory-driven conditions. Individuals living with such conditions often display a lower prevalence of traditional risk factors, nevertheless, they can show a 24% to almost three-fold increase in cardiovascular mortality risk compared with the general population. This is true for rheumatoid arthritis (RA) (9, 10), systemic lupus erythematosus (SLE) (11, 12), severe psoriasis (13, 14), inflammatory bowel disease (IBD) (15, 16), human immunodeficiency virus (HIV) infection (17, 18), cancer survivors (19–21), and virtually any low-grade inflammation-associated disorder (22). And each presents unique challenges and considerations for risk stratification highlighting the need for more comprehensive assessment strategies.

Furthermore, stratification of inflammatory-driven risk is largely unexplored in primary and secondary (residual risk) prevention levels. In individuals classified as low-risk or high-risk, in primary prevention, traditional scores generally perform well. However, they may miss those with non-traditional risk factors, subclinical disease or in contemporary cohorts with improved cardiovascular prevention strategies (e.g., widespread use of statins, smoking cessation programs, and better blood pressure control) (23, 24). Moreover, traditional scores are least effective in the intermediate-risk group where a proportion of over 50% of individuals are misclassified and can be re-stratified when additional biomarkers are considered (25–28). In secondary prevention, estimates show that 38% of individuals persist with elevated high-sensitivity C-reactive protein (CRP) and these patients exhibit higher all-cause mortality, myocardial infarction and stroke rates at one year compared with those with lower CRP (29, 30). In this review we aim to gather and appraise studies presenting alternatives to traditional risk assessment that consider the inflammatory contribution to cardiovascular events in primary and secondary prevention levels, as well as, in populations living with inflammatory-driven diseases.

## Methods

**Figure 1:**
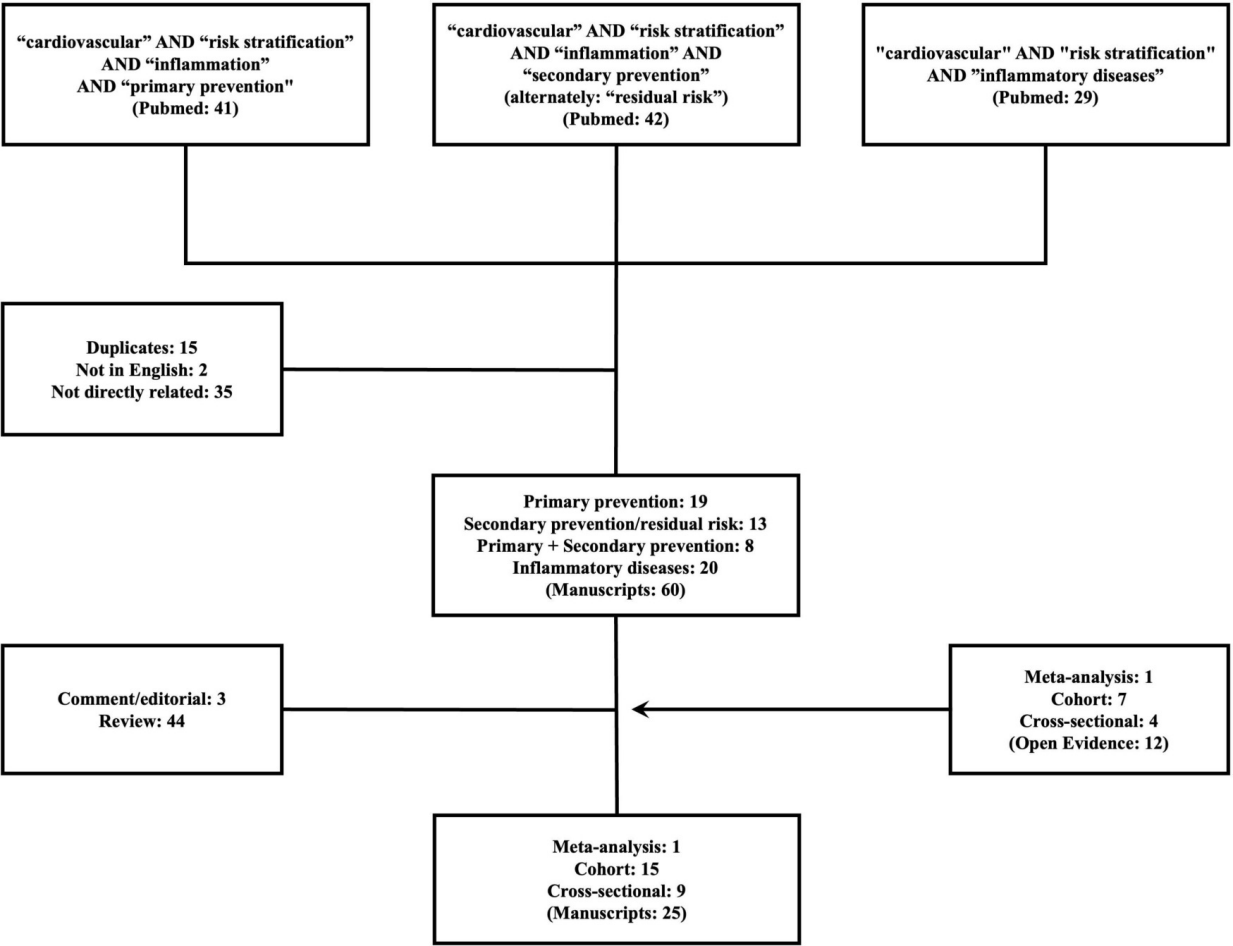
Flowchart outlining the selection of studies reviewed and critically appraised.

Two researchers (TQ, GLRS) entered the search terms: “cardiovascular” AND “risk stratification” AND “inflammation” AND alternately the following aditional terms: “primary prevention” OR “secondary prevention” OR “residual risk” OR “inflammatory disease” in the PubMed platform. The search period was set until March 10th, 2025. Manuscripts with unavailable full texts, that were not in English or were not directly related to the topic of this review were excluded (Figure 1). Cross-sectional and prospective cohorts presenting survival analyses were given priority to the purpose of this review. In parallel, Open Evidence, a medical large language model platform, was assessed to provide supplementary texts through standardized prompts, as follows: “What are the validated scores/tools to assess the effect of inflammation on cardiovascular events in primary prevention?”, “What are the validated scores/tools to assess the effect of inflammation on cardiovascular events in secondary prevention?”, and “What are the scores/tools to assess the cardiovascular risk in patients with inflammatory diseases?”. Follow-on questions were posed as deemed appropriate by both researchers. Additional relevant manuscripts were accessed by a manual search through the references of the initially retrieved studies to put data in context. All additional papers were included when there was agreement between researchers.

## Results

A total of 112 manuscripts were initially found through Pubmed. Forty were excluded: 35 (31.2%) did not meet the scope of the study and 15 (13.4%) were duplicates. Also, 47 (41.9%) manuscripts did not provide original data and were also excluded: 3 (2.6%) were comment/editorial and 44 reviews. Thirteen (11.6%) studies from Pubmed were considered for the present scoping review: 8 cohorts (7.1%) and 5 (4.5%) cross-sectional studies. Additional research through Open Evidence retrieved 12 articles that were also added: 1 meta-analysis, 7 cohorts and 4 cross-sectional studies. In total, 25 studies were analysed: 1 meta-analysis, 15 cohort studies, 9 cross-sectional studies and discussed throughout the text (Tables 1–3). Other studies were included to support the evidence and provide context.

**Table 1:** Validated cardiovascular risk scores that incorporate inflammatory markers in primary prevention.

Risk score	Validated population	Year of validation	Outcomes evaluated	Accuracy	Components	Prevention level
Reynolds Risk Score (28, 97)	Healthy adults (initially women, then men)	2007 (Women), 2008 (Men)	Incident CVD events (MI, stroke, revascularization, CVD death)	C-statistic: 0.72–0.78 (depends on sex).	Age, SBP (Systolic Blood Pressure), total cholesterol, HDL-C (High-Density Lipoprotein Cholesterol), hs-CRP, family history of MI, aspirin use	Primary
Systemic Inflammation Response Index (SIRI) (98)	Cancer and CVD patients	2022	One-year cardiac mortality, percutaneous intervention, and CABG	C-statistic: 0.72–0.87 (+ improves traditional risk model)	(Neutrophil count × Monocyte count)/Lymphocyte count. This index reflects the balance between pro-inflammatory (neutrophils, monocytes) and anti-inflammatory (lymphocytes) immune cell populations.	Both
Systemic Inflammatory Index (SII) (45, 98, 99)	CVD and cancer patients	2022, 2024	All-cause mortality, heart failure, non-fatal myocardial infarction	C-statistic: 0.59–0.78 RR: 1.87–2.32	(Platelet count × Neutrophil count)/Lymphocyte count. Elevated SII suggests increased inflammation and potential thrombotic risk.	Both
Systemic Inflammation Immunity Index (SIIRI) (100, 101)	Malignancies and CVD patients	2023, 2025	Cardiovascular death, nonfatal myocardial infarction, and nonfatal stroke	C-statistic: 0.72–0.79	(Neutrophil count × Platelet count × Monocyte count)/(Lymphocyte count). This index further refines the assessment by considering the interplay of multiple immune cell types.	Both
CHA2DS2-VASc with Inflammatory Biomarkers (102)	Atrial fibrillation (AF) patients	2010 (CHA2DS2-VASc), later	Stroke, systemic embolism, mortality	Improves C-statistic of CHA2DS2-VASc	Original CHA2DS2-VASc components (Congestive heart failure, Hypertension, Age ≥75, Diabetes mellitus, Stroke/TIA/thromboembolism, Vascular disease, Age 65–74, Sex category) + inflammatory biomarkers (e.g., high-sensitivity troponin T, IL-6).	Both
Inflammatory Prognostic Scoring (IPS) (103)	Adults with hypertension	2022	Cardiovascular and all-cause mortality	C-statistic: 0.69-0.74 (10-year)	LDH, CRP, ALP, WBC, NEU, LYM, Mno, RDW, red cell distribution width; NLR, dNLR, MLR, SIRI.	Primary

CABG, coronary artery by-pass graft surgery; CVD, cardiovascular disease; MI, Myocardial Infarction; LDH, lactate dehydrogenase; hs-CRP, (high-sensitivity C-reactive protein); ALP, alkaline phosphatase; WBC, white blood cell; NEU, neutrophils; LYM, lymphocyte; Mno: monocyte; RDW, red cell distribution width; NLR, neutrophil-to-lymphocyte ratio; dNLR, derived neutrophil-to-lymphocyte; MLR, monocyte-to-lymphocyte ratio; SIRI, systemic inflammatory response index; TIA, transient ischemic attack.

**Table 2:** Validated cardiovascular risk scores in secondary prevention (not all include inflammatory biomarkers).

Risk score	Validated population	Year of validation	Outcomes evaluated	Accuracy	Components	Prevention level
PREDICT-CVD Secondary Prevention Score (49)	Established CVD (MI, stroke, PAD)	2017	CVD events (MI, stroke, CVD death, heart failure hospitalization)	C-statistic: 0.72–0.73	Age, sex, ethnicity, smoking, DM, heart failure, PAD, prior MI/stroke, SBP, total/HDL cholesterol, eGFR, medications (statins, ACEi, aspirin)	Secondary
GRACE 2.0 Score (104)	Acute Coronary Syndrome (ACS)	2021	In-hospital mortality, long-term mortality, MACE	C-statistic: 0.640–0.754 (1-year follow-up)	Age, heart rate, SBP, creatinine, Killip class, cardiac arrest, ST-segment deviation, elevated troponin, prior MI	Secondary
TIMI Risk Score for Secondary Prevention (TRS 2P) (105, 106)	Established ASCVD (MI, stroke, PAD)	Late 2000s/Early 2010s	MACE (MI, stroke, CVD death)	C-statistic: 0.68–0.69	Age, hypertension, diabetes, current smoking, heart failure, peripheral artery disease, prior MI/stroke	Secondary
PREDICT-ACS Risk Score (50)	Acute Coronary Syndrome (ACS)	2020	ACS, HF, stroke, peripheral vascular disease or a CV death	C-statistic: 0.68–0.70	Age, heart rate, SBP, Killip class, cardiac arrest, ST-segment deviation, elevated troponin, eGFR, prior MI/stroke	Secondary
Glasgow Prognostic Score (GPS) (51, 52)	Cancer patients (initially), then CVD patients	2004 (Cancer), 2025 (CVD)	Overall survival, CVD events	HR for GPS: 1) 1.66; 2) 2.75	CRP (C-reactive protein), albumin	Secondary
GRACE Score with ML Enhancements (107)	ACS patients (STEMI, NSTEMI, unstable angina)	2023	In-hospital mortality, long-term mortality, MACE	Improves C-statistic of original GRACE score	Original GRACE components (age, heart rate, SBP, creatinine, Killip class, cardiac arrest at admission, ST-segment deviation) + inflammatory biomarkers (selected by ML)	Secondary

ACS, acute coronary syndrome; CVD, cardiovascular disease; HDL, high density cholesterol; HF, heart failure; MI, Myocardial Infarction; MACE, major adverse cardiovascular events; ML, machine learning; SBP, systolic blood pressure; PAD, peripheral artery disease; TIA, transient ischemic attack.

**Table 3:** Validated or adapted cardiovascular risk scores for autoimmune diseases or chronic low-grade inflammatory conditions.

Risk score	Validated population	Year of validation	Outcomes evaluated	Accuracy	Components	Prevention level
SLICC/ACR Risk Score (108)	Systemic Lupus Erythematosus (SLE)	2002	Survival or late mortality	RR: 1.5–14.4	Age, sex, ethnicity, smoking, HTN, DM, lipids, renal function, SLE SLEDAI, cumulative organ damage, antiphospholipid antibodies (aPL), medications (corticosteroids, HCQ)	Both
Framingham Risk Score (FRS) Modified for Rheumatoid Arthritis (RA) (109, 110)	Rheumatoid Arthritis (RA)	Mid-2000s onward	CVD events (MI, stroke, CVD death)	C-statistic: 0.74–0.82; 1.5 factor multiplier	Standard FRS components + RA-specific factors (disease duration, RF/anti-CCP positivity, extra-articular manifestations) + inflammatory markers (ESR, CRP)	Both
D:A:D 2016 model (111)	People Living with HIV (PLWH)	2016-onward	MI, stroke, death, ICP	C-statistic: 0.71–0.83	Conventional risk factors + CD4 lymphocyte count, cumulative protease inhibitor and NRTI exposure, and current abacavir use	Both
PCE Modified for Chronic Kidney Disease (CKD) (112)	Chronic Kidney Disease (CKD)	Mid-2010s onward	MI, stroke, CVD death, heart failure	Improved C-statistic vs. standard PCE	Standard PCE components + CKD-specific factors (eGFR, UACR)	Both
Modified Framingham Risk Score (mFRS) for SLE (75)	Systemic Lupus Erythematosus (SLE)	2016	Non-fatal; MI or stroke, and CVD deaths	C-statistic: 0.67–0.78, 2.0 factor multiplier	Age, sex, total cholesterol, HDL cholesterol, systolic blood pressure, treatment for hypertension, smoking status, and SLE (added risk)	Primary
SLECRISK tool (113)	Systemic Lupus Erythematosus (SLE)	2024	Non-fatal; MI or stroke, and CVD deaths	C-statistic: 0.69–0.80	ACC/AHA Risk Score + Disease Activity at Last Visit, Disease Duration, Creatinine, Anti-dsDNA, Anti-RNP, Lupus Anticoagulant, Anti-Ro, Low C4	Primary

CKD, chronic kidney disease; CVD, cardiovascular disease; CVE, Cardiovascular Events; BMI, body mass index; eGFR, estimated glomerular filtration rate; HDL, high density cholesterol; ICP, invasive cardiovascular procedures; FRS, Framingham Risk Score; MI, Myocardial Infarction; MACE, major adverse cardiovascular events; PCE, pooled cohort equations; ML, machine learning; SBP, systolic blood pressure; PAD, peripheral artery disease; SLE, systemic lupus erythematosus; SLEDAI, SLE disease activity; TIA, transient ischemic atack; UACR, urine-to-creatinine ratio.

### Historical context and advancements

The understanding of inflammation's role in ASCVD has evolved substantially over the past few decades, transitioning from a first recognition of association to a more nuanced understanding of causal mechanisms. Early epidemiological studies, notably the Physician's Health Study and the Women's Health Study, demonstrated a strong association between elevated levels of inflammatory markers, such as CRP, and an increased risk of future cardiovascular events, including myocardial infarction and stroke (31). These seminal findings sparked intense research efforts aimed at finding and validating other inflammatory biomarkers that could improve ASCVD risk prediction and provide insights into the underlying pathophysiology.

Initially, the research focus was primarily on acute-phase reactants like CRP and interleukin-6 (IL-6), which are readily measurable and reflect systemic inflammatory activity. However, as research progressed, it became evident that a broader spectrum of inflammatory mediators, including cytokines (e.g., TNF-α, IL-1β), chemokines (e.g., MCP-1), and adhesion molecules (e.g., ICAM-1, VCAM-1), also contribute to the pathogenesis of ASCVD by modulating immune cell recruitment, endothelial activation, and vascular remodeling (32).

More recently, the concept of inflammaging was introduced. It is characterized by a persistent elevation of circulating pro-inflammatory cytokines (such as IL-6, TNF-α, and CRP), activation of innate immune pathways (notably the NLRP3 inflammasome), and dysregulation of both adaptive and innate immunity. The process of inflammaging not only contributes to atherosclerosis itself but also interacts with conventional cardiovascular risk factors amplifying their adverse cardiovascular effects (33). Mechanistically, it involves a deteriorated macrophage and lymphocyte function leading to an increase in neutrophil-to-lymphocyte ratio. Also contributing is a surge of inflammatory proteins, growth factors, and enzymes that senescent cells (cells that permanently stopped dividing) secrete. They are collectively known as secretory-associated senescense phenotype (SASP). The deteriorated immune system does not clear SASP efficiently. Moreover, mitochondrial dysfunction and oxidative stress, age-related changes in the gut microbiome (dysbiosis) and impaired autophagy, together with the accumulation of damage-associated molecular patterns (DAMPs) and pathogen-associated molecular patterns (PAMPs)—molecules from damaged cells or from microorganisms—complete the mechanistic setting of age-associated inflammation (34). These factors drive a pro-inflammatory milieu that accelerates vascular aging, endothelial dysfunction, and atherosclerosis, thereby increasing the risk of myocardial infarction, stroke, heart failure, and other age-related cardiovascular diseases (35).

Clonal hematopoiesis of indeterminate potential (CHIP) is also increasingly recognized as a driver of systemic low-grade inflammation with substantial mechanistic overlap with inflammaging (36). The presence of CHIP confers a 2–4-fold increase in the incidence of coronary artery disease (37). The mutation arises from age-related somatic mutations in hematopoietic stem cells—most commonly in DNMT3A, TET2, ASXL1, and JAK2—leading to clonal expansion of mutant leukocytes (36). These mutant cells, particularly monocytes and macrophages, exhibit a pro-inflammatory phenotype, characterized by increased secretion of cytokines such as IL-1β, IL-6, and TNF-α, and activation of the NLRP3 inflammasome pathway, which is central to both CHIP-driven and age-related sterile inflammation (38, 39). Strategies under investigation to potentially mitigate its cardiovascular effects include senolytics to clear senescent cells, NLRP3 inflammasome inhibitors, modulation of the gut microbiome, and lifestyle interventions such as diet and exercise, which have demonstrated efficacy in reducing systemic inflammation and improving cardiovascular outcomes (40).

The development of sophisticated imaging techniques, such as coronary computed tomography angiography (CCTA), positron emission tomography (PET), and magnetic resonance imaging (MRI), has further enhanced our ability to assess subclinical atherosclerosis and inflammation *in vivo*. These imaging modalities allow for the non-invasive detection and characterization of coronary artery plaque burden, composition (e.g., lipid-rich necrotic core), and inflammatory activity (e.g., 18F-FDG uptake), providing valuable information for ASCVD risk stratification and monitoring treatment response. For instance, CCTA can identify high-risk plaques with features such as low attenuation and positive remodeling, which are associated with an increased risk of future cardiovascular events (41).

### Primary prevention cardiovascular risk stratification tools

#### Traditional risk assessment models

Traditional ASCVD risk assessment models, such as the Framingham Risk Score, the Pooled Cohort Equations, the Predicting Risk of Cardiovascular Disease Events (PREVENT) and the European SCORE system, are widely used in clinical practice to estimate the 10-year risk of cardiovascular events in asymptomatic individuals (42). These models primarily rely on conventional risk factors such as age, sex, blood pressure, total cholesterol, HDL cholesterol, smoking status, and diabetes. While these tools are valuable for identifying individuals at high risk of ASCVD in the general population, they often underestimate risk in patients with inflammatory-driven conditions, leading to delayed or inadequate preventive interventions (24).

**Figure 2:**
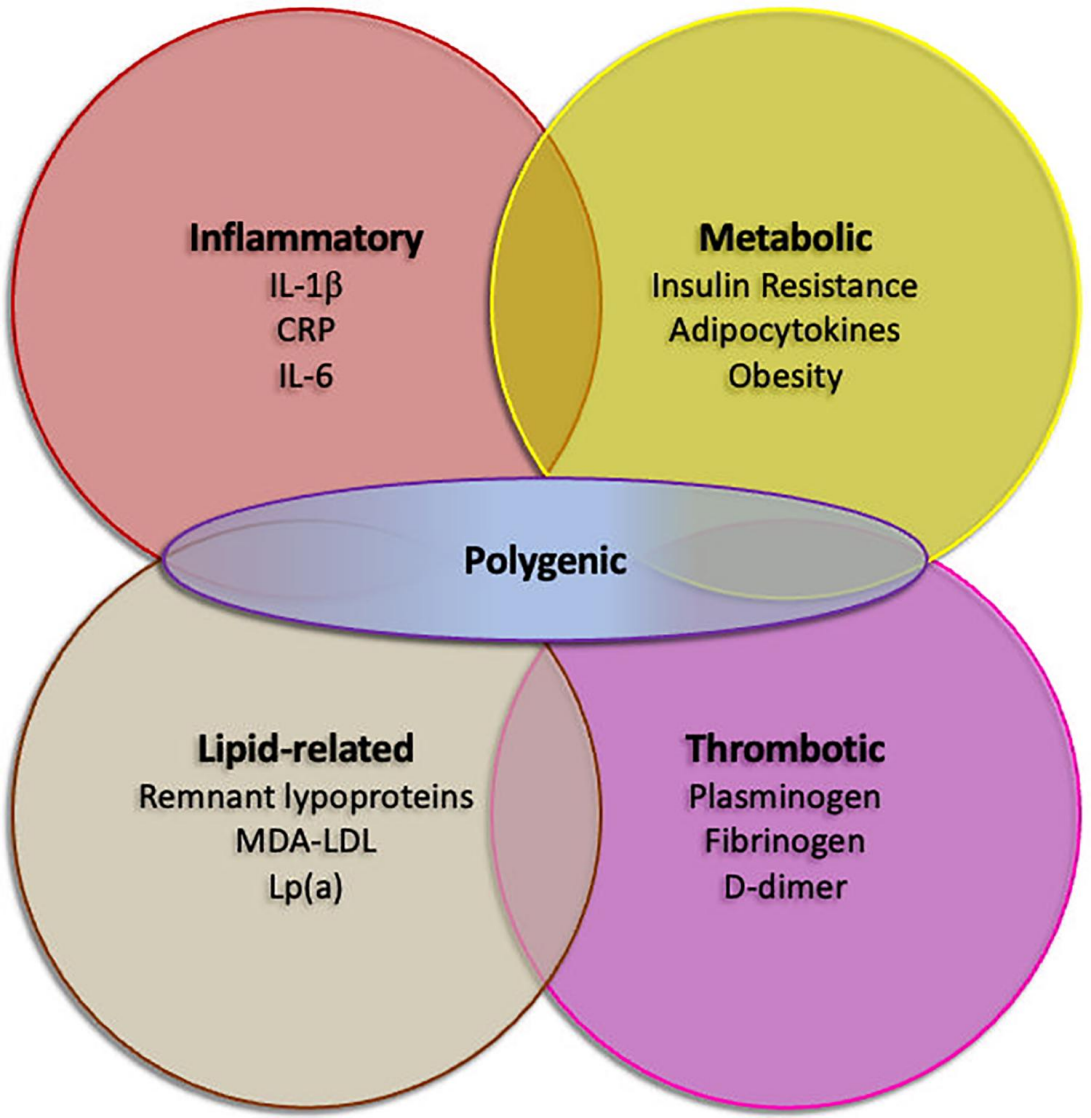
Residual risk factors in secondary prevention despite optimal guideline-directed medical therapy. Inflammatory, lipid-related, thrombotic and metabolic factors are recognized as possible contributors of recurrent cardiovascular events. Polygenic risk, as assessed by polygenic risk scores, may possibly enhance any of the factors. CRP, C-reactive protein; IL-1β, interleukin 1β; IL-6, interleukin 6; Lp(a), lipoprotein(a); MDA-LDL, malondialdehyde-modified low density lipoprotein.

The limitations of traditional risk assessment models in these populations stem from their failure to adequately account for the independent and additive contribution of inflammation to ASCVD risk, as well as other components of residual risk (Figure 2). Patients with chronic inflammatory diseases or infections often have persistently elevated levels of inflammatory biomarkers, which can accelerate atherosclerosis and increase the likelihood of cardiovascular events, even in the absence of traditional risk factors or with well-controlled traditional risk factors (23). For example, a young woman with systemic lupus erythematosus (SLE) may have a seemingly low FRS score based on conventional risk factors but still be at high risk for ASCVD due to chronic inflammation and immune dysregulation.

In particular, CRP has emerged as a valuable marker for assessing residual inflammatory risk in patients with established ASCVD. The JUPITER trial, a landmark study, showed that statin therapy reduced cardiovascular events in individuals with elevated CRP levels (≥2 mg/L), even in the absence of hyperlipidemia, providing strong evidence for the benefit of targeting inflammation in secondary prevention (43).

#### Tools incorporating inflammatory biomarkers in primary prevention

To address the limitations of traditional risk assessment models, researchers have explored the incorporation of inflammatory biomarkers into ASCVD risk stratification algorithms. Several studies have investigated the incremental predictive value of adding inflammatory markers, such as CRP, IL-6, lipoprotein-associated phospholipase A2 (Lp-PLA2), and other novel biomarkers, to conventional risk scores (28). Some of these validated risk assessment tools are available despite not being widely adopted (Table 1).

The Reynolds Risk Score (RRS) is one prominent example of a risk assessment tool that incorporates CRP in addition to traditional risk factors. Studies have shown that the RRS improves ASCVD risk prediction, particularly in women and younger individuals, and may be particularly useful for identifying high-risk individuals who are not adequately classified by traditional risk scores (28). In some cohorts, CRP was a stronger predictor than LDL cholesterol (31). The QRISK2 score, commonly used in the UK, also incorporates measures of chronic kidney disease, ethnicity, renal disease, atrial fibrillation, and rheumatoid arthritis which can indirectly reflect inflammatory burden and genetic predisposition to ASCVD. QRISK2 demonstrated superior calibration and discrimination compared to the Framingham score (44).

The systemic immune-inflammation index (SII) may be a potential marker for ASCVD development. An elevated SII is associated with an increased risk of ASCVD. However, the quality of evidence is generally low. The overall pooled results showed that higher SII was significantly associated with an increased risk of ASCVD (HR = 1.39, 95%CI: 1.20–1.61, *P* < 0.001). This increased risk could be observed in almost all ASCVD subtypes, including ischemic stroke (HR = 1.31, 95%CI: 1.06–1.63, *P* = 0.013), hemorrhagic stroke (HR = 1.22, 95%CI: 1.10–1.37, *P* < 0.001), myocardial infarction (HR = 1.11, 95%CI: 1.01–1.23, *P* = 0.027), and peripheral arterial disease (HR = 1.51, 95%CI: 1.18–1.93, *P* = 0.001) (45). The Inflammatory Prognostic Scoring (IPS) was specifically developed to stratify risk of adults with hypertension. Demographic characteristics and 12 inflammatory markers were assessed for prognostication power. Lactate dehydrogenase, alkaline phosphatase, lymphocyte count, neutrophil-to-lymphocyte ratio, monocyte-to-lymphocyte ratio, systemic inflammatory response index (SIRI), and red cell distribution width (RDW) were the strongest predictors. Time-independent ROC analysis revealed an optimal IPS cut-off value of 0.482 for 5-year [sensitivity: 73.1%; specificity: 64.6%; AUC = 0.747 (0.716–0.779)], and 0.402 for 10-year [sensitivity: 71.4%; specificity: 61.7%; AUC = 0.709 (0.682–0.737)] prediction of cardiovascular mortality.

While some studies have shown a statistically significant improvement in risk prediction, others have reported only modest or inconsistent benefits. Conventional biomarkers might not be sufficient, highlighting the need to identify novel, more specific markers. Furthermore, the optimal inflammatory biomarker (or panel of biomarkers) and the appropriate cut-off values for risk stratification have not been definitively established, limiting the widespread adoption of these tools in clinical practice (46). Thus, the clinical utility of incorporating inflammatory biomarkers into ASCVD risk stratification remains a subject of ongoing debate and investigation.

The effectiveness and clinical utility of ASCVD risk stratification tools incorporating inflammatory biomarkers depend on several factors, including the specific biomarker used, the population studied, the clinical context, and the availability of effective interventions to target inflammation. While some studies have shown that these tools can improve risk prediction and guide treatment decisions, others have raised concerns about their cost-effectiveness, potential for over-treatment, and the lack of robust evidence supporting the use of specific anti-inflammatory therapies for primary prevention (47). One of the major challenges in implementing inflammatory biomarker-based risk stratification is the lack of standardization in biomarker assays and the variability in biomarker levels over time. Different assays may yield different results, making it difficult to compare data across studies and to set up universal cut-off values for risk stratification. Moreover, inflammatory biomarker levels can be influenced by various factors, such as acute infections, trauma, medications (e.g., statins, NSAIDs), and lifestyle factors (e.g., smoking, obesity), which can complicate their interpretation in clinical practice (47).

Despite these challenges, there is growing evidence that incorporating inflammatory biomarkers into ASCVD risk assessment can be particularly useful in certain high-risk populations, such as patients with chronic inflammatory diseases, a strong family history of premature ASCVD, or metabolic syndrome. In these individuals, the presence of elevated inflammatory markers may warrant more aggressive risk factor management, including lifestyle modifications, statin therapy, and consideration of novel anti-inflammatory therapies, as well as closer monitoring for cardiovascular events (48).

### Residual cardiovascular risk stratification in secondary prevention

#### Approaches for stratifying residual risk

Secondary prevention strategies, including lifestyle modifications (e.g., smoking cessation, healthy diet, regular exercise), pharmacotherapy (e.g., statins, antiplatelet agents, ACE inhibitors), and revascularization procedures (e.g., PCI, CABG), have significantly reduced the risk of recurrent cardiovascular events in patients with established ASCVD. However, a substantial proportion of these patients continue to experience adverse events despite optimal adherence to guideline-recommended therapies (Figure 2), highlighting the critical need for improved strategies to stratify residual risk and identify individuals who may benefit from more intensive or novel interventions (29, 30).

#### Tools for risk stratification in secondary prevention

There are several risk assessment scores and tools available to evaluate residual risk in patients with established ASCVD. These tools are designed to predict future cardiovascular events and guide clinical decision-making. However, only a few have incorporated inflammatory biomarkers as predictors (Table 2). The PREDICT-CVD Secondary Prevention Score was developed to estimate short-term ASCVD risk in patients with prior ASCVD. It incorporates routine clinical measurements such as ethnicity, comorbidities, body mass index, and treatment, alongside established risk factors. It takes into account an inflammation-associated condition, renal impairment. The score was validated in both New Zealand and UK cohorts, showing good calibration and performance, although it tends to overestimate risk in patients with heart failure (49). The PREDICT-ACS, designed to predict 30-day major adverse cardiovascular events after an acute coronary syndrome (ACS), also includes renal function in its components (50). But neither study from the PREDICT framework include inflammatory biomarkers in its prediction tool.

A couple studies on secondary prevention assessed the prognostication utility of systemic inflammation markers. The Glasgow Prognostic Score (GPS) was initially validated in cancer patients as a predictor of survival. Subsequently, GPS was explored in ASCVD settings. The score is simple to calculate and utilizes readily available laboratory parameters (CPR and albumin). It provides a snapshot of the patient's inflammatory and nutritional status (51, 52). In a second study, investigators using machine learning algorithms added inflammatory biomarkers (CRP, albumin, white blood cell count) to the well-established GRACE score. ROC analysis revealed a modest increase in the AUC from 0.63 in the original score to 0.84 in the GRACE + inflammatory markers score for 6-month death/myocardial infarction prediction after an ACS. Interestingly, CRP and albumin were also among the strongest predictors of events.

#### Potential role of inflammatory markers in refining risk stratification

Inflammatory markers play an important role in refining risk stratification in patients undergoing secondary prevention, providing insights into the ongoing inflammatory processes that contribute to residual risk. Several studies have demonstrated that elevated levels of inflammatory biomarkers, such as CRP, IL-6, and TNF-α, are independently associated with an increased risk of recurrent cardiovascular events, even after adjusting for traditional risk factors and standard secondary prevention therapies (53, 54).

Over one third of individuals post-myocardial infarction remain with elevated CRP and these patients present higher all-cause mortality, myocardial infarction and stroke rates at one year compared with those with lower CRP (29, 30). However, the use of inflammatory markers in secondary prevention remains an area of active research and clinical debate. Some experts advocate for routine measurement of CRP in all patients with established ASCVD to guide treatment decisions, while others recommend a more selective approach, focusing on patients with persistently elevated risk despite optimal risk factor control or those with a history of inflammatory conditions.

Some studies have focused on developing and evaluating novel anti-inflammatory therapies that can reduce residual cardiovascular risk in patients undergoing secondary prevention. The CANTOS trial, a groundbreaking study, demonstrated that canakinumab, a monoclonal antibody that selectively targets IL-1β, significantly reduced the risk of recurrent cardiovascular events in patients with a history of myocardial infarction and elevated CRP levels (55). This trial provided the first direct evidence that targeting inflammation can improve cardiovascular outcomes in secondary prevention. The COLCOT trial also demonstrated that targeting inflammation after recent myocardial infarction can improve outcomes (56). In patients with ACS undergoing percutaneous coronary intervention (PCI), residual inflammatory risk, as indicated by elevated CRP levels, is associated with increased risks of ischemic events, cardiac death, and all-cause mortality (57). LoDoCo2 trial showed similar results in the chronic setting (58).

The Cardiovascular Inflammation Reduction Trial (CIRT) further supports the predictive value of inflammatory markers such as IL-6, IL-1β, and CRP for recurrent cardiovascular events, even though, it was a neutral trial (54). CIRT did not screen for CRP thus resulted in a median level of only 1.6 mg per liter at randomization. Also, inflammatory markers were not affected by methotrexate, the investigational drug (59). Nevertheless, baseline levels of IL-6, CRP, and LDL were all predictors of major recurrent cardiovascular events; adjusted hazard ratios [HR; 95% confidence interval (CI)] for the lowest to highest baseline quartiles of IL-6 were 1.0 (referent), 1.66 (1.18–2.35), 1.92 (1.36–2.70), and 2.11 (1.49–2.99; *P* < 0.0001), while adjusted HRs for increasing quartiles of CRP were 1.0 (referent), 1.28 (0.92–1.79), 1.73 (1.25–2.38), and 1.79 (1.28–2.50; *P* < 0.0001). Effect estimates were not statistically different in these analyses for pairwise comparisons between IL-6, CRP, or LDL, although IL-6 was the strongest predictor of all-cause mortality (59).

Altogether, these studies reinforce the role of inflammatory biomarkers in stratifying residual risk, although not all assessed these markers at randomization. Also, that the risk of cardiovascular events may depend on specific inflammatory pathways. Inhibition of IL-1β–IL-6 signaling, a process initiated at the level of the NLRP3 inflammasome seem to be implicated in atherothrombosis (60). Other anti-inflammatory drugs tested have resulted neutral studies and did not change any of the above biomarkers (61, 62). Research exploring other markers like the neutrophil-to-lymphocyte ratio and lipoprotein(a) are ongoing.

### Cardiovascular risk assessment in patients with inflammatory diseases

**Figure 3:**
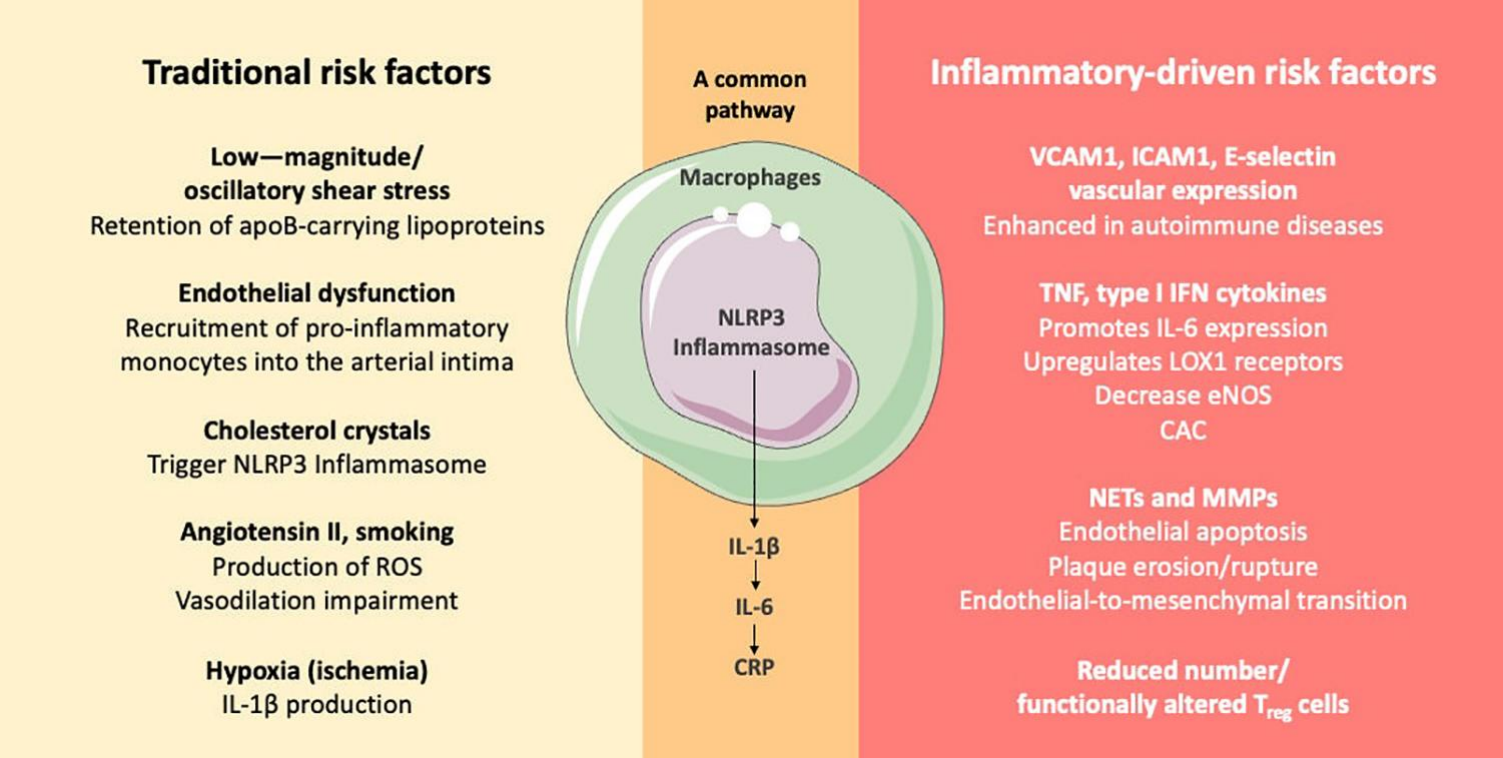
Didactic representation of specific atherogenesis pathways associated with traditional and with inflammatory-driven risk factors. Autoimmune conditions, such as SLE and RA, enhance cardiovascular inflammatory pathways comprising innate and adaptive immunity further contributing to atherogenesis (64). apoB, apolipoprotein B; CAC, coronary artery calcification; CRP, C-reactive protein; eNOS, endothelial nitric oxide synthase; MMP, matrix metalloproteinase; NET, neutrophil extracellular trap; ICAM1, intercellular adhesion molecule 1; IFN, interferon; IL-1β, interleukin 1β; IL-6, interleukin 6; LOX1, lectin-type oxidized LDL receptor 1; Regulatory T cell, Treg cells; ROS, reactive oxygen species; TNF, tumor necrosis factor; VCAM1, vascular cell adhesion molecule 1.

The inflammatory pathways involved in cardiovascular events in the general population and those in patients with inflammatory autoimmune diseases share similarities but also exhibit distinct characteristics (Figure 3). In both groups, inflammation is a critical component of cardiovascular risk, with chronic inflammation being linked to the development and progression of atherosclerosis and other ASCVD. In the general population, inflammation is recognized as an independent risk factor for ASCVD, with pathways involving cytokines such as IL-1, IL-6, and TNF playing significant roles in atherogenesis and plaque instability (63). Targeting these pathways has shown promise in reducing cardiovascular risk, as evidenced by studies on IL-1 and IL-6 inhibition (55, 63).

In patients with inflammatory autoimmune diseases, such as rheumatoid arthritis and systemic lupus erythematosus, the risk of ASCVD is markedly increased. The inflammatory mechanisms in autoimmune diseases often involve aberrant leukocyte function, reduced or functionally altered T_reg_ cells, pro-inflammatory cytokines, such as type 1 interferon, and autoantibody production, which contribute to vascular dysfunction and chronic inflammation (64, 65). These mechanisms overlap with those seen in the general population but are often more pronounced due to the underlying autoimmune pathology. Furthermore, immune-mediated inflammatory diseases can lead to a proatherogenic dyslipidemic state, characterized by dysfunctional HDL and increased LDL oxidation, which further exacerbates cardiovascular risk (66). The impact of disease-modifying antirheumatic drugs on lipid profiles and cardiovascular risk is complex and varies by specific disease and treatment regimen (66).

Overall, while there are shared inflammatory pathways between the general population and patients with autoimmune diseases, the latter group experiences additional risk due to the specific immunopathogenic features of their conditions. This highlights the need to tailored approaches to cardiovascular risk management that address both traditional risk factors and the unique inflammatory processes present in autoimmune diseases (67, 68).

#### Tools for risk stratification in patients with inflammatory diseases

The European League Against Rheumatism (EULAR) emphasize the importance of regular screening and management of modifiable ASCVD factors. Several risk assessment methodologies have been proposed and developed for patients with inflammatory diseases, incorporating disease-specific variables and biomarkers (Table 3). However, according to these recommendations, due to the lack of validated disease-specific tools, the use of generic prediction tools is advisable (69).

Common cardiovascular risk prediction tools like QRISK-3, Framingham Risk Score, and Reynolds Risk Score have been evaluated in patients with inflammatory conditions such as rheumatoid arthritis, psoriatic arthritis, and ankylosing spondylitis. These tools often underperform in these populations, suggesting the need for disease-specific risk prediction tools. QRISK-3, for instance, tends to overpredict cardiovascular risk, while Framingham Risk Score and Reynolds Risk Score may underpredict it, especially in high-risk individuals. Studies evaluated the predictive performance of the Framingham Risk Score, QRISK2, Systematic Coronary Risk Evaluation (SCORE), Reynolds Risk Score, American College of Cardiology/American Heart Association Pooled Cohort Equations, Expanded Cardiovascular Risk Prediction Score for Rheumatoid Arthritis (ERS-RA), and the Italian Progetto CUORE score. Efforts to include nontraditional risk factors, disease-related variables, multipliers, and biomarkers largely failed to substantially improve risk estimates (70, 71).

The Systemic Coronary Risk Evaluation (SCORE) for RA (SCORE-RA) was developed to better estimate ASCVD risk in patients with rheumatoid arthritis. This score incorporates rheumatoid arthritis-specific factors, such as disease duration, rheumatoid factor positivity, anti-cyclic citrullinated peptide (anti-CCP) antibody positivity, and functional disability, in addition to traditional risk factors (72). Rheumatoid arthritis-specific factors contribute significantly to cardiovascular risk in RA patients, 30% of ASCVD events are attributable to these characteristics (73).

A few cardiovascular risk assessment tools have been evaluated in predicting ASCVD events in patients with SLE. These include QRISK2, QRISK3, Framingham Risk Score, modified Framingham Risk Score for SLE (mFRS), and the SLE Cardiovascular Risk Equation (SLECRE). SLECRE, while having the highest sensitivity, has the lowest specificity. Among these, the mFRS has been found to be superior to the Framingham Risk Score and comparable to QRISK tools. The mFRS is considered a practical tool for ambulatory settings due to its simplicity and adjustment for SLE (74). The mFRS applies a two times multiplier to the Framingham Risk Score for a patient with an SLE diagnosis (75).

In patients with psoriatic arthritis, an increased cardiovascular risk has also been observed. EULAR suggests using a multiplication factor of 1.5 for cardiovascular (CV) risk algorithms in patients with inflammatory arthritis. However, studies have shown that adapting CV risk algorithms according to EULAR recommendations did not improve their discriminative ability or calibration in psoriatic arthritis patients. Commonly used tools include SCORE, CUORE, FRS, QRISK2, and Reynold's Risk Score, but these tools often underestimate or overestimate CV risk in rheumatic patients (71, 76).

Cancer and cardiovascular disease share similar conventional risk factors such as hypertension, diabetes, smoking, age, among others, and that systemic inflammation is related to the progression of both clinical conditions (77). CANTOS trial additionally provided insights into the potential role of IL-1β inhibition in reducing lung cancer incidence and mortality, as well as its effects on other inflammatory conditions (55). With the advancement of oncological therapies and greater survival rates, especially in higher incidence cancers such as breast and prostate, mortality from cardiovascular disease may exceed mortality from cancer itself, according to data from an American cohort (78). Despite the important role of systemic inflammation in this population there is a scarcity of validated ASCVD risk scores in the oncological setting and they usually do not include systemic inflammation biomarkers (79).

### Implementation of inflammatory risk assessment in cardiovascular disease prevention according to guidelines

In primary prevention, CRP should not be used for routine cardiovascular risk assessment according to the European Society of Cardiology (ESC) guidelines (80). The use of CRP is best reserved for cases where traditional risk factors do not provide clear guidance, and its measurement should not replace established risk algorithms such as SCORE2 (ESC) or the Pooled Cohort Equations (AHA/ACC) (80, 81). The American Heart Association (AHA) and American College of Cardiology (ACC) guidelines endorse selective use of CRP in primary prevention for individuals at intermediate risk (10%–20% 10-year risk) when the decision to initiate statin therapy is uncertain. In these cases, CRP measurement may help refine risk estimates and guide therapy. However, the AHA/ACC position is cautious, assigning CRP a class IIb recommendation (may be considered), and does not support its use for routine screening or serial monitoring (82). Additionally, recent evidence highlights that apparently healthy individuals, especially women without standard modifiable risk factors but with elevated CRP (“SMuRF-less but inflamed”), may also benefit from biomarker assessment, as they are at increased risk for incident cardiovascular events and may be missed by conventional risk algorithms (83). The incremental value of CRP is less pronounced in low-risk (<10% 10-year risk) or high-risk (>20% 10-year risk) individuals.

The landscape shifts considerably in secondary prevention, where inflammatory risk assessment takes on greater clinical significance. In patients taking contemporary statins, CRP was a stronger predictor for future cardiovascular events and death than LDL-C (84, 85). Elevated CRP levels in patients taking statins and PCSK9 inhibitors may indicate residual inflammatory risk that could be further reduced through inflammation modulation (86). Furthermore, experimental inhibition of interleukin-6, a pivotal factor in atherothrombosis, resulted in a marked parallel reduction of CRP and fibrinogen in patients with chronic kidney disease and high cardiovascular risk (87). These findings support the emerging paradigm that inflammation represents a distinct therapeutic target in patients who have achieved optimal lipid control but continue to demonstrate elevated inflammatory markers.

The complexity of cardiovascular risk assessment is amplified in patients with chronic inflammatory conditions. Patients with human immunodeficiency virus (HIV) are twice as likely to develop ASCVD compared with the general population (17). In people living with HIV, the Infectious Diseases Society of America now recommends statin therapy for all individuals aged ≥40 years, regardless of baseline LDL-C or calculated ASCVD risk, following the REPRIEVE trial's demonstration of a 36% reduction in major cardiovascular events with pitavastatin (88). This practice-changing recommendation reflects the inadequacy of traditional risk scores in this population and the heightened risk conferred by chronic immune activation. The American Heart Association also recognizes HIV infection as an ASCVD risk enhancer and supports consideration of subclinical imaging (e.g., coronary artery calcium scoring, carotid ultrasound) and selected biomarkers (CRP, Lp(a), apoB) for further risk stratification in ambiguous cases (89).

Similarly challenging are patients with rheumatic diseases. Identification of ASCVD through carotid artery plaque screening may be considered in ASCVD and CAD risk evaluation in these patients (90–92). Standard risk calculators tend to underestimate true risk due to the impact of chronic inflammation and disease-modifying therapies. Therefore, adjunctive modalities such as carotid ultrasound for intima-media thickness and plaque detection are recommended to improve risk stratification, particularly in women and those with atypical lipid profiles (93). Emerging surrogate markers (e.g., paraoxonase activity, endocan, osteoprotegerin) and advanced imaging techniques may further refine risk assessment, though their routine use is not yet established in guidelines (94).

The rationale for enhanced screening recommendations in inflammatory conditions is that chronic inflammation accelerates atherosclerosis and increases ASCVD risk, often leading to underestimation of risk by standard calculators. Therefore, clinicians should consider these conditions as risk-enhancing factors and may initiate moderate-intensity statin therapy if the estimated 10-year ASCVD risk is ≥5%, after optimizing lifestyle interventions for 3–6 months (95). For patients with rheumatoid arthritis, rechecking lipid values 2–4 months after achieving disease control is advised, as inflammation can mask dyslipidemia (95). The guidelines also note that since accuracy of standard risk estimators is limited, additional tools such as coronary artery calcium (CAC) scoring may be considered if statin therapy decisions remain uncertain (95). The justification is that inflammation, immune activation, and certain therapies (e.g., glucocorticoids, antiretrovirals) further elevate risk and alter lipid metabolism, necessitating tailored screening and management strategies. Overall, systematic screening in these populations should be more frequent, multimodal, and personalized, integrating both traditional and disease-specific risk factors, and leveraging imaging and biomarkers where appropriate.

### Emerging risks and impact of specific treatments

Emerging risks in ASCVD risk assessment in patients with inflammatory diseases include the recognition of novel inflammatory mediators, the impact of emerging therapies, and the role of the microbiome in modulating inflammation and ASCVD risk. For example, recent studies have identified new inflammatory biomarkers, such as neutrophil extracellular traps (NETs), microRNAs (miRNAs), and inflammasome activation products, that may contribute to ASCVD pathogenesis in these populations (96). These novel biomarkers may provide additional insights into the mechanisms linking inflammation and ASCVD and offer potential targets for therapeutic intervention.

The development of new therapies for inflammatory diseases, such as Janus kinase (JAK) inhibitors (e.g., tofacitinib, baricitinib) and interleukin inhibitors (e.g., secukinumab, ustekinumab), has also raised questions about their long-term impact on ASCVD risk. While some studies have suggested that these therapies may have cardioprotective effects by reducing systemic inflammation, others have raised concerns about potential adverse cardiovascular outcomes, such as increased risk of venous thromboembolism and major adverse cardiovascular events. Post-marketing surveillance studies and long-term clinical trials are needed to fully evaluate the cardiovascular safety of these emerging therapies (64).

It is essential to carefully monitor patients with inflammatory diseases for emerging ASCVD risks and to tailor treatment strategies to minimize their overall ASCVD burden. This may involve optimizing traditional risk factor management, using cardioprotective medications (e.g., statins, ACE inhibitors), carefully considering the potential cardiovascular effects of specific therapies for inflammatory diseases, and promoting healthy lifestyle behaviors. Furthermore, emerging evidence suggests that modulating the gut microbiome through dietary interventions or fecal microbiota transplantation may have beneficial effects on inflammation and ASCVD risk in patients with inflammatory diseases.

## Conclusion

Cardiovascular risk stratification in inflammatory-driven conditions has advanced in recent years, fueled by a deeper understanding of the multifaceted role of inflammation in ASCVD pathogenesis. While traditional risk assessment models have inherent limitations in these populations, the incorporation of inflammatory, disease-specific factors, and advanced imaging techniques into risk scores has shown promise in improving risk prediction and guiding clinical decision-making.

One of the major challenges in ASCVD risk assessment in patients with inflammatory diseases is the inherent heterogeneity of these conditions. Inflammatory diseases can manifest with a wide range of clinical presentations, disease activity levels, associated comorbidities (e.g., renal disease, anemia), and genetic backgrounds, making it difficult to apply a one-size-fits-all approach to risk assessment and management. Moreover, the lack of standardization in biomarker assays, the complex interplay of treatment-related effects, and the need for more targeted and personalized anti-inflammatory therapies are remaining challenges on the field. Future research should focus on developing more refined and individualized approaches to ASCVD risk stratification and management in patients with inflammatory conditions, taking into account their unique risk profiles, disease characteristics, and treatment history. The discovery and validation of novel biomarkers and imaging techniques, as well as the rigorous evaluation of emerging therapies, will further refine the ability to prevent cardiovascular events and improve long-term outcomes in these high-risk populations.
